# How should communities be meaningfully engaged (if at all) when setting priorities for biomedical research? Perspectives from the biomedical research community

**DOI:** 10.1186/s12910-022-00879-5

**Published:** 2023-02-06

**Authors:** Josephine Borthwick, Natalia Evertsz, Bridget Pratt

**Affiliations:** 1 Royal Australian College of General Practitioners, Melbourne, Australia; 2 Royal Melbourne Hospital, Melbourne, Australia; 3 Queensland Bioethics Centre, Australian Catholic University, 1100 Nudgee Rd., Brisbane, Australia; 4 Centre for Health Equity, School of Population and Global Health, University of Melbourne, Melbourne, Australia

**Keywords:** Ethics, Priority-setting, Engagement, Participation, Patient and public involvement, Biomedical research, Genomics research

## Abstract

**Background:**

There is now rising consensus that community engagement is ethically and scientifically essential for all types of health research. Yet debate continues about the moral aims, methods and appropriate timing in the research cycle for community engagement to occur, and whether the answer should vary between different types of health research. Co-design and collaborative partnership approaches that involve engagement during priority-setting, for example, are common in many forms of applied health research but are not regular practice in biomedical research. In this study, we empirically examine the normative question: should communities be engaged when setting priorities for biomedical research projects, and, if so, how and for what purpose?

**Methods:**

We conducted in-depth interviews with 31 members of the biomedical research community from the UK, Australia, and African countries who had engaged communities in their work. Interview data were thematically analysed.

**Results:**

Our study shows that biomedical researchers and community engagement experts strongly support engagement in biomedical research priority-setting, except under certain circumstances where it may be harmful to communities. However, they gave two distinct responses on what ethical purpose it should serve—either empowerment or instrumental goals—and their perspectives on how it should achieve those goals also varied. Three engagement approaches were suggested: community-initiated, synergistic, and consultative. Pre-engagement essentials and barriers to meaningful engagement in biomedical research priority-setting are also reported.

**Conclusions:**

This study offers initial evidence that meaningful engagement in priority-setting should *potentially* be defined slightly differently for biomedical research relative to certain types of applied health research and that engagement practice in biomedical research should not be dominated by instrumental goals and approaches, as is presently the case.

## Introduction

Community engagement is a core feature of participatory health research, and there is growing agreement that it is essential for all health research, from biomedical to applied forms [1]. As Nunn et al. affirm, it is estimated that by 2025, nearly two billion people worldwide will have had their DNA sequenced and this creates a global imperative for engagement in genomic research [2]. Community engagement is now encouraged, or even mandated, as a key element of all health research, including ‘traditional’ non-participatory health research, by research institutions and funding bodies [3–5]. For example, it was a required component of applications to the second and final round of funding for the H3Africa Consortium, which supports genomics research in Africa [6]. Many high-profile genomics research initiatives have made public statements about the importance of involving the community [2]. Community engagement is also increasingly required by international biomedical research ethics guidelines [5–7].

Arguments have been made that it is *ethically* essential to undertake some form of engagement in health research because it is central to showing respect for communities and the traditions and norms that they share, increases the chances that research will improve health outcomes, builds public trust, enhances prospects for justice, and facilitates better stewardship of resources [1]. Where engagement is undertaken as shared decision-making throughout the research process, it facilitates self-determination because those who are significantly affected by the selection of health research priorities and the translation of the evidence generated by their investigation are included in discussions and decision‐making about them [8]. Such engagement also promotes cognitive and epistemic justice and maximizes the social knowledge generated to identify and solve complex problems that impede health and well‐being [8]. It is seen as a key means to ensure that research projects ask the ‘right’ questions—namely, those that are responsive to urgent community-identified needs—and create ‘better’ knowledge that uses and reflects a diversity of knowledge systems and is shared beyond peer-reviewed journals and academic conferences [9].

Yet debate continues about when, how, and for what purpose engagement should be performed in health research, and whether the answer should vary between different types of health research. Several types of ethical goals have been attributed to community engagement in health research: instrumental, intrinsic, and transformative. Engagement can advance intrinsic goals like building a sense of inclusion or demonstrating respect [3]. Engagement activities can further purely instrumental goals such as facilitating smooth research operations, augmenting the efficiency of study recruitment, or gaining community ‘buy-in’ [4, 10]. It also has “the potential to redress past harms; compensate for or resolve existing differences in power, privilege, and positionality; [and] allow for marginalized voices and experiences to be represented in the production of scientific knowledge” [4, p. 257].

Who is engaged can vary considerably based on how the “community” is defined. A community can be defined based on geography; shared experiences, characteristics, or ethnicity; or special interests or goals [3]. In health research, communities are often related to the nature of the research activity, e.g., the geographical area or illness group a given study involves. Engagement activities then frequently include patients, carers, and/or the broader public. However, depending on how the relevant community is defined, ministries of health, ethics committees, policymakers, international organizations, the media, and universities may be engaged as well [3].

What constitutes community engagement in health research practice also varies dramatically along a spectrum from shallow and tokenistic engagement to deeper and more meaningful engagement. Engagement takes different forms, ranging from raising community awareness of research projects, to consultation on certain parts of research projects, to community representation throughout the entire research cycle, to long-term and authentic partnerships [11]. Where these forms of engagement fall on the spectrum largely relates to the *stage of the research cycle* at which engagement occurs, and the *level of participation* they afford to those engaged. Goulet contends that the earlier ‘non-elites’ enter the process, the higher the quality of their participation. In the health research project context, this means entry during grant writing and priority-setting comprises deeper participation than, for instance, when communities enter during data collection or analysis [12].

Even so, the quality of communities’ participation is not exclusively determined by when they enter into the research process. A range of levels of participation exist, with some more “active, deliberative, and influential” than others [13, 14]. Informing refers to generating awareness and understanding within host communities about what research is and about already defined research projects that are being undertaken with them. The outputs of decision‐making in research projects are shared with community members; they do not give their input and are not involved in making the decisions. Consulting is a particularly common form of engagement in health research [3, 15]. Community members are asked to give their input (e.g., feedback, suggestions, critiques) on aspects of research projects but with no guarantee that those who decide will use or consider the information they provide. There is broad consensus that they can potentially be consulted about a wide variety of health research activities, including protocol development, research conduct, access to data and samples, and the dissemination or publication of research findings [16]. Often, their consultation occurs through specifically established bodies such as community advisory boards.

Collaborative partnership, on the other hand, means involving community members in decision-making throughout health research projects. This is consistent with growing emphasis on practicing engagement in health research as the co‐construction of knowledge or co-design. Taking such approaches means researchers jointly construct knowledge with communities: all parties design and conduct research together in ways that achieve the purposes of both sets of actors [17]. Community members are part of assessing what local health problems should be the focus of the research; planning, conducting and overseeing the research; and integrating the research into the health care system [18]. These approaches thus share many similarities with community-based participatory action methodologies. A key point of difference between them and other forms of engagement is the balance of power. Consulting and informing do not entail community members’ participation in decision‐making, whereas collaboration does.

A key matter to investigate is whether the type of health research is ethically significant in specifying what engagement should entail—namely, when, how, and for what moral purpose engagement should be performed. A spectrum of health research disciplines exists, ranging from basic science, clinical, and genomics research to more applied types like public health, health services, and health policy research. It has been suggested that types of applied health research place a greater emphasis on meaningful engagement with communities [5]. Co-design and participatory action methods are not regular practice in genomics or other types of biomedical research [2]. Questions have been raised by researchers as to whether co-design methods are even possible in biomedical research, which, unlike public health or health services research, requires more specialised and technical knowledge [19]. A scoping review investigating public involvement in human genomics projects found their involvement was highest at the stage of “implementation and management” (19/32), while the stages of engagement with the lowest number of initiatives reporting involvement were “funding” (1/32) and “identifying topics” and “prioritization” (4/32)” [2]. Similarly, a recent realist review showed that the collaborative partnership thread of community engagement is less common in biomedical research. Instead, where engagement with instrumental goals and approaches dominates [20]. Thus, biomedical research potentially seems less suited and/or inclined to adopt more meaningful forms of engagement, but this does not necessarily mean they aren’t nonetheless ethically ideal.

This paper investigates what form of community engagement (if any) ideally belongs in biomedical research priority-setting. We empirically examine the question: should communities be engaged when setting priorities for biomedical research projects, and, if so, how and for what purpose? Biomedical research is defined as encompassing *basic science, clinical*, and *genomics* research. Basic science and clinical research have traditionally fallen within the biomedical research category. Over the past few decades, genomics has also become a central and cohesive discipline of biomedical research [21]. Research priority-setting refers to defining the research topic and study questions for individual health research projects or programs. It does not encompass defining a set of global, national, or institutional research topics that should receive priority funding and implementation. This study’s focus on priority-setting reflects the moral importance of engagement from the beginning of research projects. Communities’ early engagement equates to deeper participation and enhances the responsiveness of research priorities. It means community members are part of making a greater number of decisions about a given study, including those that determine the direction of the entire research project. Without engagement from priority-setting, researchers may ultimately miss the needs deemed of high import and urgency by communities [22]. Research has shown that patients, based on their lived-experience, prioritize different topics than experts [23]. Despite this, in practice, communities concerningly often do not participate in the priority-setting stage of biomedical research projects [2]. Our study’s focus, however, is not meant to suggest it is sufficient to engage communities in the priority-setting stage of health research projects alone.

We conducted in-depth interviews with 31 members of the biomedical research community from the UK, Australia, and African countries who had engaged communities in their work. Both genomics and clinical research have become increasingly globalised since the 1990s [24, 25]. As such, the recruitment of interviewees spanned both high-income countries and low- and middle-income countries. African countries were selected to provide a low- and middle-income country perspective due to the growth of clinical and genomics research initiatives requiring community engagement on the continent. The UK and Australia were selected to provide a high-income country perspective because engagement in health research is established in both countries. Since “patient and public involvement” in health research has been a feature of UK policy for longer and is widely adopted by UK research funders [26], it was also thought that UK interviewees might have different ideas and experiences related to what comprises ideal engagement in biomedical research priority-setting than Australian or African interviewees. We analysed interview data thematically and report four main themes in the paper: the value of engagement, pre-engagement essentials, ideal goals and models of engagement, and barriers to engagement in biomedical research priority-setting. We conclude by considering whether our findings suggest community engagement in priority-setting should occur but look different for biomedical research relative to applied health research, should occur and look the same, or should not occur.

## Methods

### Study methods and sample

We performed 31 semi-structured interviews with basic science, clinical, and genomics researchers who had engaged communities in their work (27 interviewees) and community engagement experts who were embedded in biomedical research (4 interviewees).In-depth interviews were chosen as the primary method to explore the research question because they allow for the rich details of key informants’ experiences and perspectives to be gathered. All procedures were performed in accordance with the National Health and Medical Research Council of Australia’s *National Statement on Ethical Conduct of Human Research*.

The sampling strategy used a mix of purposive and snowball methods. To recruit Australian interviewees, we identified potential candidates systematically through a structured search, targeting five universities in Australia with high-calibre biomedical/genomic institutes. We approached sixteen Australian researchers via email and interviewed eight. Five did not respond, and three did not believe their experience to be relevant to the research question. Another three interviewees were identified via snowball sampling.

To identify UK and African interviewees, we initially employed a similar strategy targeting UK and African universities, but it was very difficult to tell from academic profiles in those countries whether individuals had experience with community engagement in biomedical research. Across the UK and Africa, not many academics advertised that they did community engagement. As such, we revised the sampling strategy to target biomedical consortia and institutions that we knew were supportive of and/or funded by entities that require community engagement and that funded/employed UK and African biomedical researchers: the Sanger Institute (UK), the US National Institutes of Health, and the H3Africa Consortium. The Sanger Institute was chosen because its researchers spanned basic science, clinical, and genomics research and it has links to Wellcome Trust, which have funding requirements for community engagement. H3Africa was selected because it is a large consortium of genomics researchers spanning the African continent and its funding made community engagement a required component of applications [6, 27]. The NIH was chosen because its researchers were likely to have done community engagement to secure funding^1^ and it was easy to find UK and African scientists through their website. We identified the websites of NIH centres for biomedical research and searched their staff directories for UK and African professors/group leaders. At all three institutions, senior researchers were approached, with the rationale being that they could refer on junior research team members as potential interviewees. In total, we approached 195 UK researchers and interviewed seven. Another thirteen were identified via snowball sampling, of whom three were interviewed. We approached 63 African researchers and interviewed three. Another 24 were identified via snowball sampling, of whom seven were interviewed. The low success rates reflect that many potential interviewees declined due to being overburdened with clinical responsibilities during the Covid-19 pandemic.

Study inclusion and exclusion criteria are described below (Box 1). All interviews were conducted remotely. Sampling continued until no new information emerged and saturation was achieved.

**Box 1:** Interview inclusion and exclusion criteria

*Inclusion criteria*
Members of the biomedical research community who have experience with community engagement in their home country and/or overseas contexts
English-speaking
Ability to be interviewed remotely (i.e., Phone call, Zoom, Skype)
Over the age of 18
*Exclusion criteria*
Researchers or others involved in performing other types of health research
Non-English speaking
Participants who are unable to be interviewed remotely
Under the age of 18

In total, our 31 interviewees came from the UK, Australia, Kenya, South Africa, Malawi, Nigeria, Uganda, and Burkina Faso. Eighteen women and thirteen men were interviewed. Interviewees’ work spanned basic science (4), clinical (13) and genomics (17) research. Three interviewees had experience with both clinical and genomics research. Interviews’ duration was approximately 40–75 min. 22 interviewees had experience with community engagement in priority-setting. Of those, nine were from Australia, three were from Africa, and ten were from the UK.

### Data collection and analysis

We conducted semi-structured interviews according to the technique of *thick description* [28]. Thick description means that questions for interviewees are open-ended and attempt to draw out interviewees’ experiences and views. In keeping with in-depth interviewing techniques, explanatory probes (such as asking “why” and “could you tell me more about that”) were also used to elicit richer details and clarificatory probes were employed to generate a better understanding of interviewees’ comments [29]. The interview guide used in this study is provided in Box 2 below.

**Box 2:** Interview guide

What kind of biomedical research (basic science, clinical, or genomics) do you do?
Have you have engaged communities in biomedical research agenda setting? Why have you chosen to do so?
Should communities be engaged in biomedical research priority-setting? Why or why not?
What do you think community engagement in should ideally look like in biomedical research priority-setting? Who should be engaged and how?
In your experience, what is important to ensure people can raise their voices equally and be heard when engaging communities in biomedical research priority-setting?
In your experience, what barriers exist relating to meaningfully engaging communities in biomedical research priority-setting?
Is there anything you would like to share about meaningful engagement in biomedical research priority-setting that you haven’t already spoken about during the interview?

Interviews were transcribed verbatim and thematic analysis of interview data was undertaken in the following five phases: initial coding framework creation, coding, intercoder reliability and agreement assessment, coding framework modification, and final coding of entire dataset [30, 31]. Two coders (NE and JB) independently examined six transcripts and identified categories and subcategories. They then developed an initial coding framework together and discussed that framework with BP. Using the initial coding framework, NE and JB next undertook an iterative process of coding a transcript, assessing intercoder reliability and agreement, and modifying the coding framework [30]. Here, a “negotiated agreement approach” was adopted, whereby NE and JB separately coded a transcript, compared their codings, and then discussed their disagreements in an effort to reconcile them and arrive at a final version in which as many discrepancies as possible were resolved [31]. Six transcripts were co-coded. Across the six transcripts, coders ultimately agreed with proposed inclusion/exclusion of codes 100% of the time. Where a coder identified codes that the other had not, agreement to include JB’s codes occurred 92% of the time and agreement to exclude occurred 8% of the time, and agreement to include NE’s codes occurred 89% of the time and agreement to exclude occurred 11% of the time. During this process, the coding framework was modified, discussed with BP, and finalised. NE then applied the final coding framework to recode the African interviewee transcripts, and JB applied it to recode the UK and Australian interviewee transcripts. All data was coded using NVivo software. From this analysis, four main themes emerged: the value of engagement, pre-engagement essentials, ideal goals and models of engagement, and barriers to engagement in biomedical research priority-setting.

## Results

### The value of engagement

Most interviewees strongly affirmed there is value for community engagement when determining priorities for biomedical research projects: *“communities should be engaged in the conceptualisation [of research]…at the time of planning, communities at the table bring in their own perspective.” – African interviewee (06).* Interviewees described four reasons for why engagement should occur in priority-setting– epistemic value, community ownership, equity, and responsiveness (see Table 1). Interviewees across all geographical contexts (Africa, the UK, Australia) strongly affirmed that the epistemic knowledge held by community members is a critical resource for any priority-setting exercise in biomedical research. According to one UK interviewee (01), *“… the people who have the best knowledge of those problems are the people that live with particular conditions or challenges that the research is trying to benefit.”*

**Table 1:** Reasons for community engagement in biomedical research priority-setting

Reason	Example quote
Epistemic value	*“… questions which, because we’re not patients we don’t think of them in the same way whereas the patients have questions or problems that they think need addressing which we don’t necessarily think about from a medical point of view.”—UK interviewee (10)* *“I think people with lived experience… I think academics, particularly in things like genomics; we have this interest in following things that are shiny, and kinda cool. Sometimes we may lose sight of the real reason as to why we are undertaking these sorts of activities. I think by having consumer involvement in research design, we can still be aspirational about the outcomes of research. So we can still have our…head in the clouds, but it keeps out feet on the ground and really grounded in lived experience, and the reality of these conditions that we are researching.”—Australian interviewee (05)*
Community ownership	*“So they’re owning it… if you plan with them and you end up developing what they need, you’re not gonna ask them, you’re not gonna force them to use it, be it knowledge, it’s information or be it a product, it’s theirs and they use it.”—African interviewee (08)*
Equity	*“…we don’t actually engage with consumers and with communities who are supposed to benefit from it from the beginning. And to me it’s just another way that equity and inequity kind of slips into our research, that no matter how much we say this research is good and it’s gonna benefit people, unless we actually have the people who it’s supposed to benefit with onboard at the beginning it’s actually going to harm them more than it’s gonna help them.”—Australian interviewee (05)*
Responsiveness	*“In an ideal world as I said I think it is important that when a researcher decides to come up with their research idea, before actually develops their proposal, they should go to the community to find out whether that research idea will actually translate into solving a health problem that is there.”—African interviewee (02)*

However, a minority of interviewees expressed concern about carrying out community engagement during biomedical research priority-setting under specific *unsafe* circumstances. Some interviewees worried that community engagement in particular countries or generally could put individuals from certain patient communities at risk of harm. According to an African interviewee (07),

*“ [I] think the reason why we decided to leave them out it’s because homosexuality is illegal in the country, right, it’s not accepted and so sometimes if you actually involve such individuals in the [HIV] research study sometimes you put them at risk. The same with sexual, we call them sex workers yeah."*

Where a sub-group within a patient community experiences stigma or a patient community experiences a stigmatised illness, it may be ethically appropriate to instead engage family members, or individuals who are close to them, or to not engage them at all. An interviewee from Africa (07) provided an example,

*"I’ve been involved in some psychiatric genomic research as well, we know that psychiatric patients in Africa are completely marginalised right…How do you engaged those people right? Who carriers their voice? Now you can do beautiful community engagement around psychiatric illness, and we’ve tried right, we’ve even engaged patients with schizophrenia… but to get them to really help you set research priorities is, is you know what’s quite a different kettle of fish and so what often happens is you engage their family members…"*

Interviewees further highlighted that, in other contexts, it may not be culturally safe for communities to engage with researchers. As one Australian interviewee (08) described,

*“…what I am seeing is this reflex to, really rapid, I would say, quick and dirty relationship being formed, and then expected that a letter of support will be written, without the research being properly discussed with anyone beyond the leadership of a particular organisation. So, what that means is, there has often not been sufficient community consultation”*.—Australian interviewee (08).

Where a “loose threads” or tokenistic approach to engagement is taken, there is insufficient planning, inadequate time for engagement, and limited engagement occurs, often random, one-on-one communication. For example, one member of a research team speaks to one person within an Aboriginal organisation. This is not representative of a community and can reinforce unequal power dynamics within a community. Such practices may cause more harm to communities than benefit.

They are often a product of a research economy, where funders require evidence of community engagement, but pay little attention to the depth of engagement. Where there is no funding for community engagement in priority-setting, and/or no requirement (or expectation) from funders to *meaningfully* engage communities when determining research priorities.

### Pre-engagement essentials

Interviewees identified two components that comprised *pre-engagement essentials:* defining the community and building foundations*.* These should be achieved *before* attempting to engage a community in biomedical research priority-setting.

#### Defining the community

First, defining the community with whom to engage was thought to be essential in biomedical research priority-setting. Interviewees described two overlapping ways of defining the community that are common to biomedical research: *communities of people who live with a certain disease* and *communities defined by geographical boundaries*. The latter was more commonly described by African interviewees and the former by Australian and UK interviewees. Some interviewees also explained that they used geographical boundaries to define a patient community, as some diseases or genomic conditions are more prevalent in certain areas.

#### Building foundations

Four main types of foundations were thought to be essential to have in place before meaningful engagement can occur in biomedical research priority-setting: environmental, relational, collective and individual (see Table 2). Of these, none were specific to biomedical research relative to health research broadly, with the exception of genomic literacy. Genomic literacy means those engaged have a base level of understanding of genomics concepts and terms. Interviewees believed such literacy is critical to the success of genomics research priority-setting discussions involving community members. In instances where there is not an existing level of genomic literacy, efforts should be made to educate the community.

**Table 2:** Foundations for meaningful engagement

	Example quote
*Environmental*	
Time	*“Engagement to me and what I’ve seen like working with Aboriginal and Torres Strait Islander communities and an amazing colleague who works in Indigenous health, it’s actually longer and it takes a lot more time than people think it does. And so I was like yeah, we’ll do it in three months, and it’s like no, no you need eighteen months to make sure you’re doing this right.”—Australian interviewee (04)*
Organisational support and governance frameworks	*“I think community engagement does require a governance framework to be successful. Community engagement is one activity that occurs within the space of a research project in the space of collaboration. So I do think the governance framework is an essential foundation for the community engagement to occur.”—Australian interviewee (08)*
Funding requirements for community engagement	*“… So the NIHR which is the National Institute for Health Research in the UK so a bit like the NIH in the States, so it has a PPI [patient and public involvement] mantra and agenda. So in order to secure fundings from them you have to show adequate PPI.”—UK interviewee (10).* * “…that model is not around developing a suite of policies and standards, but a framework that funded research projects must adhere to, and agree to these policies if they are going to be funded. And that is around community involvement, community renumeration if appropriate and involved in publications if appropriate. So all those sorts of standards surrounding best practice in consumer involvement.”—Australian interviewee (02)*
*Relational*	
Embeddedness/existing connections to the community	*“well really since the late eighties or mid-eighties so a really, really long time… what I have realised is that you can only do this kind of sustained research (on dementia) if you have a relationship with a community.”—UK interviewee (08)* *“If you create that kind of enduring infrastructure coproduced with the community, then you have an infrastructure to identify to work with community to work with the relevant people and the community itself can identify who are the right people to be talking to about the questions which they see of, which the community identifies to be of value…”—UK interviewee (08)*
Diversity amongst the research team	*“So I think you have to, you have to be mindful of where your, your power dynamics look like in terms of—and even the kind of people you’re sending out, you know the, if you’re trying, and some of these things are unavoidable you know you might have a research group that is predominately white but they, you’ll engage for whatever reason with a different audience and already there’s a dynamic there which you have to accept that…you might not get successful engagement.”—UK interviewee (01)*
Obtaining gatekeeper support	*“What we do initially is to identify the key members of the communities who can influence the community members in making decisions. And it’s those people are usually the traditional rulers, village heads, opinion leaders. Sometimes we even involve the politicians, and the political office holders who have a strong hold in the communities. So the idea is to them first and when they get to the communities and they speak to them it’s easy to convince the members of the communities because these people are known to them, they are not strangers, they also believe that these people are interested in their wellbeing so these are the people that we think should be the stakeholders in the communities.”—African interviewee (03)*
Trust	*“So due diligence is very important, it’s very important to build trust, for people to understand what you’re doing, who you are what you’re going to do you won’t have problem.”—African interviewee (04)*
Fair processes	*“I think at the end of the day you want to make sure there is a fair playing field so the researchers and communities are working together in harmony, there is communication happening… transparency should be there as well; accountability as well…”—African interviewee (02)*
*Collective*	
Self-mobilising	*“I think it would have to be a community that is so critically aware of itself and of, of what is missing, you know what needs to be addressed to reach to that level, and it has to be a self-mobilising community… that is a really empowered community.”—African interviewee (01)*
*Individual-researchers*	
Understanding of community context	*“But in Nigeria I won’t mix male and female in Northern Nigeria, in Southern Nigeria it’s not an issue. Women speak up, men don’t override you know but in Northern Nigeria I won’t do that because when males and female are mixed up females won’t speak and then culturally you shouldn’t mix male and females.”—African interviewee (08)*
Experience with/literacy in community engagement	*“I would be interested in community engagement expertise becoming like, your biostatistics, or your statistician. It’s a given requirement that you consult with a community engagement specialist for any research that involves communities. And they contribute to the formulation of your research in terms of your objectives, your aims and your hypotheses. They help with the formulation of your ethics application, and they facilitate your approach with engaging to community. Because guess what? A lot of biomedical researchers are not necessarily skilled at engaging with communities. It is a different skill set.”—Australian interviewee (08)*
Communication skills	*“… I think it comes back to as researchers we need to do better with communication, and I think it’s putting onus on people saying that they don’t know enough, they need to learn more, we actually need to be clear with what we communicate and how we communicate and the way we do.”—Australian interviewee (11)*
Attitudes (respect for communities, recognition of value of community expertise, open-mindedness to different points of view)	*“…my research is led by people who see a value in community engagement and they’ve had a really great experience with consumer advocates as well and really found that they can give great insights and interpretations, even of the data like as we’re writing it up and putting together the manuscript.”—Australian interviewee (09)*
*Individual-community*	
Able to take a broad perspective	*“…necessary people who have a broad enough perspective to kind of be able to represent the community as a, as a bigger community…” African interviewee (09)*
Research literacy, including genomics literacy	*“…somebody who’s, who knows enough about what you’re doing to contribute but also understands the scientific processes a bit.”—UK interviewee (02)*
Known status within the community	*“So the first thing for someone to be a community consultant… they need to be known within the community those people who have a say within the community, that is one thing.”—African interviewee (06)*
Communication skills and compassion	*“So they’d also need to be good communicators, they would need to be compassionate you know all those sorts of things that you would look for, for somebody who truly represents a community.”—African interviewee (05)*

When the community of interest is defined by geographical boundaries or is marginalised, being embedded in the community is an especially important relational foundation. Identifying and obtaining the support of community gatekeepers (i.e., individuals or groups who have significant influence in the community) is a key first step to build relational foundations with such communities. Gatekeepers can facilitate access to members of geographical communities through different stakeholder groups, including local leaders and community health workers, civil society organisations, local government health committees, and/or community advisory boards. Identifying gatekeepers or community leaders is also important because they may have a *“a bigger understanding of the health needs at a national level, or they can be able to advise what the implications are at, at the national level and if it fits within their planning at that level”—African interviewee (04).*

### Ideal goals and models of engagement in biomedical research priority setting

Interviewees described two models of engagement in setting biomedical research priorities that correspond to different ethical goals: *empowerment* and *instrumental*. Interviewees cautioned against tokenistic engagement at all costs. Some interviewees believed that if engagement is not evident from research conceptualisation, it can be tokenistic:

*“You know, having a fully developed and finalised document that they wave under a community advisory group’s nose. That really just doesn’t cut it. If it’s not involvement from the start, it’s tokenistic and it’s insulting”*—Australian interviewee (05).

Having empowerment goals means community engagement assists with breaking down power disparities between community members and biomedical researchers. Several interviewees believed the ideal engagement model to achieve this goal is where communities’ initiate engagement and set research priorities, as this action can facilitate power sharing in the relationship. According to African interviewee (01), *“An ideal would be the communities reaching out to researchers to tell them what they want researchers to do. It wouldn’t be researchers engaging communities about the research researchers want to do.”* The interviewee further noted that, while the *community-initiated model* is “ideal”, self-aware and mobilising communities are rare.

Other interviewees felt empowerment goals are best furthered when research priorities arise out of synergistic relationships between communities and researchers. The *synergistic model* is embodied in an *“approach of action-based research where we would engage people at the start, and co-design research programs”* (Australian interviewee 08). In this model, community members were commonly described as “partners” and as critical members of the research team. Priority setting comprises a two-way process that requires both researchers and community members to identify research priorities*.* Where communities alone set priorities and design research, the potential for research to actually address those priorities is constrained, as the methods to address their priorities is limited to the community’s experience or imagination. Similarly, where researchers set priorities completely in isolation from those who have lived experience with a disease, they may favour inappropriate priorities and methods. Several interviewees reflected that community engagement is most powerful when it fosters an exchange of perspectives and knowledge*.*

Having instrumental goals means community engagement helps ensure that research reflecting priorities identified by researchers is more feasible. Here, researchers initiate engagement and approach a community with a research idea before they develop a research proposal. Engagement either functions to assess whether the community believes the research idea addresses a priority and, if so, to refine the idea, or it primarily functions to refine a researcher-initiated idea with the community. The former approach is described by an interviewee*:*

*“Before the researcher develops their research protocol, they should go to the communities and find out whether that particular research idea will address the priority that the communities that will be involved have. The researcher should have input from the communities at that point and then from there then they, the researcher, can draw up the research proposal and then you know, submit it for review and all that.”*—African interviewee (02).

In contrast, another interviewee suggests the latter approach

*“We’ve identified this problem in the community, for example, from preliminary reports we are seeing that there is a high level of maybe of schistosomiasis, it’s a common disease that is prevalent in communities here that really along the shores of the lakes … we tell them what kind of plan we have as the design as of the study in our mind that this is what we want to do … And then ask them for their opinions, is it feasible; do you think it’s going to be beneficial to the community”*—African interviewee (04).

Generally, instrumental goals were more commonly described as achieved through consultative models of engagement, where community members provide “input” and “information” and are “asked for opinions”.

Irrespective of whether interviewees believed that meaningful engagement should facilitate empowerment or instrumental goals, they collectively thought consideration of diversity, bringing out voices, and the nature of the engagement space are necessary in biomedical research priority-setting. Engagement of patient and geographic communities should ensure diversity in terms of what demographics are represented and, in geographic communities, what community roles are represented. Interviewees highlighted that ensuring there is ethnic diversity in biomedical research is important to capture issues affecting unique genomics populations.

Engagement should also be designed to draw out different voices, with a particular focus on marginalised voices. Interviewees describing several strategies to do so when engaging in person such as providing those engaged with background materials and pre-readings in advance; breaking into smaller groups of people with similar life-experiences; using deliberative, individualised communication; and making the engagement mirror an informal meeting. The latter reflects that “formal and structured” engagement processes can make community members uneasy, an observation more commonly reported by African interviewees.

In some circumstances, online engagement methods were recommended. The diaspora of a patient community can be reached through online engagement methods (i.e., online discussions). When considering online engagement methods, interviewees highlighted that it is important to be aware that this can disadvantage some individuals from being able to participate as they may have limited access to the necessary tools to participate (i.e., computer, internet access).

In relation to space, interviewees felt that the space chosen for engagement can influence which voices are the loudest, and which voices are missing from priority setting process. They believed that the researchers should ideally go to the community and use safe community spaces that are not imbued with exclusionary norms. Interviewees from Africa more commonly reported on the impact of cultural norms in spaces for engagement.

### Barriers to and challenges within engagement

Interviewees identified environmental, relational and individual barriers to engaging communities in setting priorities for biomedical research projects (see Table 3) and challenges that arise when doing so. Certain challenges—undefined communities, literacy, bias, and sidelining—were unique or more likely to occur in relation to engagement in genomics or biomedical research than applied health research. Interviewees reflected that one of the biggest challenges for exploratory genomics work can be the identification of the community:

*“The biggest challenge with genomics research is that it’s still a developing area and some terms you can’t identify a community or who you need to engage with kind of until you start understanding what’s going on”.*—Australian interviewee (11).

**Table 3:** Barriers to meaningful engagement

	Example quotes
*Environmental*	
Lack of resourcing/funding	*“Sometimes the budgets are so tight that to do a heap of engagement and workshops and surveys, or whatever it may be to gain community input is a barrier.”—Australian interviewee (01)* *“Ideally, we would have that approach of action-based research where we would engage people at the start, and co-design research programs. I would love to have the opportunity to do that, but I think it is challenging in terms of the way that our funding structure is set up.”—Australian interviewee (05)*
Lack of organisational support	*“I am also talking about the organisation, and the executive of the organisation, because the group leader and the researcher can end up being in a very compromised position, if what they are doing is not actually supported by, resourced, understood, invested in by the organisation at the executive level.”—Australian interviewee (08)*
Funding bodies control the agenda	*“…but you see the problem is who sets the agenda? You know sometimes it has something to do with funders or sponsors of research, they are the ones sometimes who are trying to set the agenda because I think most of our local researchers decide to develop proposals based on some cause that come from you know the sponsors or the funders like the NIH have a call for this particular topic, right, and then you know a researcher from a developing country would decide to draw up a proposal to respond to that cause.”—African interviewee (02)*
Laws	*“One of the barriers for participation in that research was that a number of the members of the communities said, “can you guarantee that, as researchers, that the genomic data you collect will not be used by the police or the government for law enforcement of other reasons?” And you know what—the researchers couldn’t guarantee that. They actually couldn’t. Because the law states that the police can have access if they request it. Right. Now that is a huge problem if you are a member of a community that is the victim of system, sustained racism for generations and is still going.”—Australian interviewee (01)*
*Relational*	
Mistrust	*“… we’ve also come across you know communities thinking that, that we are doing something as an ill will to their health and also there have been communities that can think there is some sort of black magic involved… social ways of thinking and cultural perspective is something that has to be taken into account.”—UK interviewee (12)*
Unfair power dynamics	*“…this is context specific to biomedical research, would often come from the North with the global South, that’s the context most of the time and that’s because of the money involved in this research is huge and we don’t get to take that in our local context. And that you see that in a lot of examples continue to talk about the difference and reflections, but one of the things I think happens also when you have a global North engaging with a global South there’s the power to write the narratives tend to be written by the global North.”—African interviewee (08)*
Stigma	*“I mentioned the examples of psychiatric patients, people with, with diseases that attract superstitious, or superstition so cleft lip palate for instance you know that, that general, generally people think about that as being devil’s disease…discrimination and inequality within communities is a huge barrier to meaningfully engaging those that are discriminated or marginalised…”—African interviewee (07)*
*Individual*	
Researchers unsupportive of meaningful engagement / have low levels of literacy in engagement	*“the training and researchers have, traditionally, hasn’t had anything to do with how to interact with the public or participants. It has just been how to analyse the data and produce publications.”—Australian interviewee (02)* *“I think sometimes there can be a reticence to include the community because, ‘they just won’t understand’, you know? It is a quite a paternalist perception though, isn’t it?”—Australia interviewee (05)*
Target population not wanting to engage	*“some people just don’t like interacting with their healthcare system. Usually men of my age. I don’t know why that is but you know most men my age never see their GP so they ain’t gonna engage with you. And that’s why diseases like prostate cancer don’t probably get as much attention as breast cancer.”—UK interviewee (10)*
Burden of participation	*“Because a lot of the time, when you want to talk to people. If we are talking about people who are part of the genetic and undiagnosed rare disease community, many of them are struggling day-to-day to be living with what it is they are living with. Maybe they have a family member who has got a condition. So to then be asking them to spend extra time, to be involved with agenda-setting and participating in research, and all of those kind of things, I think that can be a challenge as well.”—Australian interviewee (05).* *“There a huge under-representation of ethnic minorities and there’s a huge under representation of poorer and lower socioeconomic classes. Cos they can’t take time off from work, they’re not really interested, they’ve got no spare money.”—UK interviewee (10)*
Previous negative experiences with researchers	*“I think certainly their experiences of not being listened to make them quite reticent to actually talk.”—UK interviewee (02)*
Bias	*“… and so you do get these weird sorts of things which certainly I think would impact on, on something like if you used a patient support group to lobby you could get very biased lobbying, and priority setting you know which wouldn’t necessary be to everybody’s benefit”—African interviewee (09)*

Efforts to build genomics literacy also come with challenges

“So one of our challenges in genomics is of course that we don’t have a vocabulary and a language for genomics in many of our African settings. So that has been interesting in itself and as part of this bigger project that I’m involved in…people are actually looking at words and concepts…and how they can try and make it more accessible and also more culturally sensitive”—African interviewee (05).

Biases can arise in biomedical research priority-setting due to disease-based lobbying from different stakeholder groups and government bodies. This can result in priorities being selected for funding that are not always priorities that the community deems most important. Rare diseases can be sidelined in biomedical research priority-setting in favour of diseases that are slightly more common within a geographic community in order to address the needs of a greater number of people. But, as one interviewee affirms, “*I think you also can’t always go well if it’s common you have to call it a priority and if it’s rare you ignore it.”—African interviewee (09).* Priority-setting for rare diseases is also affected by the number of different rare disease groups competing for money. In some instances, the *“really emotive, or effective advocacy groups”* (Australian interviewee 01), may get more funding. This was not identified as a “good or a bad thing”, but the interviewee cautioned it is important to be aware of these factors. Other interviewees highlighted strategies to manage bias such as (1) undertaking fair processes that achieve transparency and accountability and (2) understanding a given community and ensuring a diversity of participants from it. These strategies were not discussed in relation to disease-based lobbying but may be relevant to dealing with it.

Certain barriers varied by location or country context. Australian and African interviewees more commonly described barriers created by unsupportive local funding structures. In contrast, achieving adequate community engagement is often required and supported by UK funding agencies. Barriers related to unfair power dynamics, especially those grounded in coloniality, were reported more often by interviewees from Africa. They commented that an impact of biomedical research funding coming from the global North to the global South means the global North holds more power over the global South, and therefore controls the narrative and elevates voices of “those they want to hear”. This can be hugely disempowering.

Stigma associated with different diseases was also commonly reported by interviewees from Africa as affecting the scope of community engagement in biomedical research. Living with certain illnesses can be hugely stigmatising for community members, and they are, therefore, often excluded from engagement generally and during priority-setting processes.

## Discussion

Whether the type of health research is ethically significant in specifying what engagement should entail is a key question to investigate. This study gathered empirical evidence that can help inform an answer. We interviewed biomedical (largely clinical and genomics) researchers and community engagement experts embedded in biomedical research from Australia, the UK, and several African countries to obtain their perspectives on whether community engagement should occur in priority-setting for biomedical research projects and, if so, how and for what purpose.

The researchers we interviewed strongly affirmed that engagement should occur as early as the priority setting phase of biomedical research projects, except under circumstances where engagement puts individuals or communities at risk of harm, and the risk cannot be mitigated. Their endorsement of early engagement is consistent not only with literature on engagement in applied and participatory health research [8, 32–35] but also with literature on engagement in genomics research [36, 37], in biomedical research [20, 38], and international biomedical research ethics guidelines [5, 39]. For instance, Ogurin et al. propose a four-stage model for community engagement in genomics research, where the first two stages include the process of conceptualising and defining the research question. Their model was developed based on interviews and focus groups with biomedical researchers, community rulers, opinion leaders, community health workers, and prospective research participants in Nigeria [36].

Study participants’ reasons for endorsing engagement during biomedical research priority-setting (see Table 1) also align with the findings of prior studies that speak to the value of early engagement in health research—namely, responsiveness, epistemic benefits, and community/local ownership or self-determination [8, 32, 40–42]. However, these and other studies identify reasons to value engagement that were not voiced by study participants here, including building relationships, opening doors, making those engaged feel valued, and giving them competence and confidence [32, 41]. This perhaps reflects the fact that our interviewees did not include those who had been engaged in health research.

Although most interviewees felt strongly that engagement should occur in biomedical research priority-setting, they gave two distinct responses on what ethical purpose it should serve. Some proposed engagement should have *transformative* goals, whereas others suggested *instrumental* goals. Both ethical goals have previously been described in the research ethics literature on engagement [4, 43] and debate continues over whether or not transformative goals should apply to engagement in health research. In this study, interviewees proposed two overlapping models of engagement as the ideal means to achieve empowerment goals: community-initiated and synergistic. The community-initiated model aligns more with Arnstein’s “citizen control” level of participation, whereas the synergistic model corresponds more closely with collaborative partnership and co-design approaches [14, 18]. Thus, the two models call for different levels of participation for those engaged.

In accordance with these findings, existing literature on engagement in genomics research also supports its being empowering and synergistic. May et al. purport that the techniques of community-based participatory research, which emphasise true partnership, should be applied in genomic science [44]. They affirm that such techniques can empower communities and can provide meaningful strategies to build trust, especially where underrepresented groups are engaged [44]. Watson et al.’s conceptual model for engagement in genomics research calls for an approach of “collaborative decision-making, facilitating dialogue, balancing power” that encompasses the priority-setting phase [37, p. 1]. Similarly, literature on participation [45–47], engagement in health research priority setting [19, 41], community-based participatory research [32], and co-design in health research [35, 48] all purport that transformative goals, collaborative partnership, and shared-decision-making are ideal or necessary to achieve more meaningful engagement.

In contrast, other interviewees in our study felt that engagement in biomedical research priority-setting should seek to achieve instrumental goals using a consultative model. Their views are consistent with some of the existing ethics literature on what engagement should look like in biomedical research [15, 49, 50], though that literature does not discuss the priority-setting phase specifically. In relation to genomics research, including priority-setting, Ogurin et al. also argue for meaningful community engagement as a way to ensure the success of a research program [36].

Although no consensus existed amongst interviewees on what the ethical goal(s) and model of community engagement should be in biomedical research priority-setting, identifying instrumental goals and consultative models as ideal is perhaps less common than in many forms of applied health research, which tends to associate meaningful engagement with empowerment goals and co-design/synergistic models. Thus, this study offers initial evidence that meaningful engagement in priority-setting should *potentially* be defined slightly differently for biomedical research relative to applied health research. Empowerment and instrumental goals achieved by community-initiated, synergistic, or consultative models may each comprise meaningful engagement in biomedical research priority-setting, though more research is needed to further assess this, both conceptually and empirically (as discussed further below in relation to study limitations). Future work should determine whether robust ethical or philosophical arguments can be made for defining meaningful engagement more broadly in biomedical research. It should also consider whether the different goals and approaches should apply under different circumstances of biomedical research priority-setting. If they should vary by context, it is necessary to determine when/where different goals and models should be used, i.e., what contextual factors demand certain goals and models of engagement. Future research could usefully further investigate the rationale for why the community-initiated or synergistic model should be used to advance empowerment goals in biomedical research priority-setting and which (if either) is better at achieving them in practice. In this study, for example, epistemic arguments were made by biomedical researchers for relying on the synergistic model.

It is also important to note that our interviewees’ endorsement of empowerment goals and models contrasts with much current biomedical research practice, where the collaborative partnership thread of community engagement is less common and instrumental goals and approaches dominate [20]. Thus, this study offers initial evidence that engagement in biomedical research priority-setting, and more broadly, should not be dominated by instrumental goals and approaches. A different balance may be ethically appropriate than what is currently practiced.

Our study identified several individual and collective qualities of researchers and community members, as well as relational and environmental essentials to build if biomedical researchers want to engage meaningfully with communities in priority-setting. Our study also identified numerous personal, relational, and environmental barriers to assess for (and address) before commencing and/or during engagement. Many of these foundations and barriers are not specific to engagement in biomedical research or during the priority-setting phase. They have been identified in previous work on engagement in health research priority-setting [51, 52] and in the wider literature on participatory development and participatory health research [32, 53–57]. However, certain barriers are likely to be more common in certain country contexts. This study suggests funding structures that are unsupportive towards engagement in priority-setting are more common in African countries and Australia relative to the UK. Unequal power dynamics and stigma were emphasised more strongly by African interviewees as barriers to engagement, which is unsurprising given the context in which much health research occurs and has historically occurred in Africa (with the global North providing funding to the global South and controlling the agenda).

Some of the challenges identified in this study are unique to engagement in *genomics* research: identifying the community and genomic literacy. This result is support by the findings of Manafo et al. and Stauton et al., who affirm that “defining ‘community’ is challenging and can depend on the particular social, cultural and geographical context in which the [genomics] research takes place.” ([58, 59], p. 2).

It is critical to acknowledge the main limitations of this study. First, this study did not solicit the voices of research participants or their communities on whether, how and for what purpose they should be engaged in biomedical research priority-setting. As a matter of epistemic justice and democratizing knowledge within the ethics field, this is a key group to focus upon in subsequent research on this topic. Second, while interviewees spanned basic science, clinical, and genomics research, few respondents to our recruitment efforts were basic scientists. This perhaps reflects the fact that community engagement in such research is less common and thus there were not as many researchers who met our inclusion criteria. Future ethics research should also solicit the views of basic science researchers on the topic of community engagement in biomedical research priority-setting. Such research could also potentially seek out the perspectives of biomedical researchers who have not engaged communities in their work. Although this study only sought the views of those researchers with some community engagement experience, other biomedical researchers would still have views about whether community engagement should happen in priority-setting or not, though they would not be grounded in actual engagement experience. Third, interviewees were recruited from Australia, several African countries, and the UK only. While engagement in biomedical research is increasingly common in these countries, there are other countries where engagement is frequently occurring in biomedical research, including non-English speaking countries and countries in other regions. Future research should capture their views as well.

## Conclusions

Our study shows that members of the biomedical research community support engagement in biomedical research priority-setting. However, interviewees did not demonstrate consensus on what ethical purpose it should serve or how it should be done. Some conveyed support for engagement to promote empowerment via co-design and community-initiated approaches, which are more common in forms of applied health research. Others endorsed a more instrumental consultative approach that is consistent with current biomedical research engagement practice. This finding suggests that how meaningful engagement in biomedical research priority-setting is defined should *potentially* look different to engagement in applied research priority-setting and that engagement should be undertaken differently to current practice. Going forward, there is still much more ethics research to do to further explore whether community engagement in priority-setting should look different in biomedical research relative to applied health research.

## Data Availability

The datasets used and/or analysed during the current study are available from the corresponding author on reasonable request.

## Abbreviations

CIOMS: Council for International Organizations of Medical Sciences
NIH: National Institutes of Health
UK: United Kingdom
UNAIDS: Joint United Nations Programme on HIV/AIDS

## Footnotes

1 The NIH is supportive of community engagement overall and has formalised it into their strategy for improving research translation. See https://www.ncbi.nlm.nih.gov/pmc/articles/PMC5582944/; https://www.niehs.nih.gov/news/assets/docs_a_e/community_engagement_efforts_at_nih_examining_best_practices_to_bridge_community_and_research_agendas_508.pdf; https://ncats.nih.gov/engagement; https://datascience.nih.gov/community-engagement.
